# Sleep slow-wave oscillations trigger seizures in a genetic epilepsy model of Dravet syndrome

**DOI:** 10.1093/braincomms/fcac332

**Published:** 2022-12-17

**Authors:** Mackenzie A Catron, Rachel K Howe, Gai-Linn K Besing, Emily K St. John, Cobie Victoria Potesta, Martin J Gallagher, Robert L Macdonald, Chengwen Zhou

**Affiliations:** Department of Neurology, Vanderbilt University Medical Center, Nashville, TN 37232, USA; Department of Neuroscience Graduate Program, Vanderbilt University Medical Center, Nashville, TN 37232, USA; Department of Neurology, Vanderbilt University Medical Center, Nashville, TN 37232, USA; Department of Neurology, Vanderbilt University Medical Center, Nashville, TN 37232, USA; Department of Neurology, Vanderbilt University Medical Center, Nashville, TN 37232, USA; Department of Neurology, Vanderbilt University Medical Center, Nashville, TN 37232, USA; Department of Neurology, Vanderbilt University Medical Center, Nashville, TN 37232, USA; Department of Neuroscience Graduate Program, Vanderbilt University Medical Center, Nashville, TN 37232, USA; Department of Neurology, Vanderbilt University Medical Center, Nashville, TN 37232, USA; Department of Neuroscience Graduate Program, Vanderbilt University Medical Center, Nashville, TN 37232, USA; Department of Neurology, Vanderbilt University Medical Center, Nashville, TN 37232, USA; Department of Neuroscience Graduate Program, Vanderbilt University Medical Center, Nashville, TN 37232, USA

**Keywords:** sleep, slow-wave oscillations, genetic epilepsy, Dravet syndrome, spike–wave discharges

## Abstract

Sleep is the preferential period when epileptic spike–wave discharges appear in human epileptic patients, including genetic epileptic seizures such as Dravet syndrome with multiple mutations including SCN1A mutation and GABA_A_ receptor γ2 subunit Gabrg2^Q390X^ mutation in patients, which presents more severe epileptic symptoms in female patients than male patients. However, the seizure onset mechanism during sleep still remains unknown. Our previous work has shown that the sleep-like state-dependent homeostatic synaptic potentiation can trigger epileptic spike–wave discharges in one transgenic heterozygous *Gabrg2^+/Q390X^* knock-in mouse model.^1^ Here, using this heterozygous knock-in mouse model, we hypothesized that slow-wave oscillations themselves *in vivo* could trigger epileptic seizures. We found that epileptic spike–wave discharges in heterozygous *Gabrg2^+/Q390X^* knock-in mice exhibited preferential incidence during non-rapid eye movement sleep period, accompanied by motor immobility/facial myoclonus/vibrissal twitching and more frequent spike–wave discharge incidence appeared in female heterozygous knock-in mice than male heterozygous knock-in mice. Optogenetically induced slow-wave oscillations *in vivo* significantly increased epileptic spike–wave discharge incidence in heterozygous *Gabrg2^+/Q390X^* knock-in mice with longer duration of non-rapid eye movement sleep or quiet–wakeful states. Furthermore, suppression of slow-wave oscillation-related homeostatic synaptic potentiation by 4-(diethylamino)-benzaldehyde injection (*i.p.*) greatly attenuated spike–wave discharge incidence in heterozygous knock-in mice, suggesting that slow-wave oscillations *in vivo* did trigger seizure activity in heterozygous knock-in mice. Meanwhile, sleep spindle generation in wild-type littermates and heterozygous *Gabrg2^+/Q390X^* knock-in mice involved the slow-wave oscillation-related homeostatic synaptic potentiation that also contributed to epileptic spike–wave discharge generation in heterozygous *Gabrg2^+/Q390X^* knock-in mice. In addition, EEG spectral power of delta frequency (0.1–4 Hz) during non-rapid eye movement sleep was significantly larger in female heterozygous *Gabrg2^+/Q390X^* knock-in mice than that in male heterozygous *Gabrg2^+/Q390X^* knock-in mice, which likely contributes to the gender difference in seizure incidence during non-rapid eye movement sleep/quiet–wake states of human patients. Overall, all these results indicate that slow-wave oscillations *in vivo* trigger the seizure onset in heterozygous *Gabrg2^+/Q390X^* knock-in mice, preferentially during non-rapid eye movement sleep period and likely generate the sex difference in seizure incidence between male and female heterozygous *Gabrg2^+/Q390X^* knock-in mice.

## Teaser

Catron et al. report that increased slow-wave oscillations cause epileptic activity in one *Dravet* syndrome mouse model with *Gabrg2^+/Q390X^* mutation. Meanwhile, the suppression of slow-wave oscillation-related synaptic plasticity *in vivo* dramatically inhibits epileptic activity in the het *Gabrg2^+/Q390X^* mice, suggesting that slow-wave oscillations can trigger seizures during non-rapid eye movement sleep.

## Graphical Abstract

**Graphical abstract:**
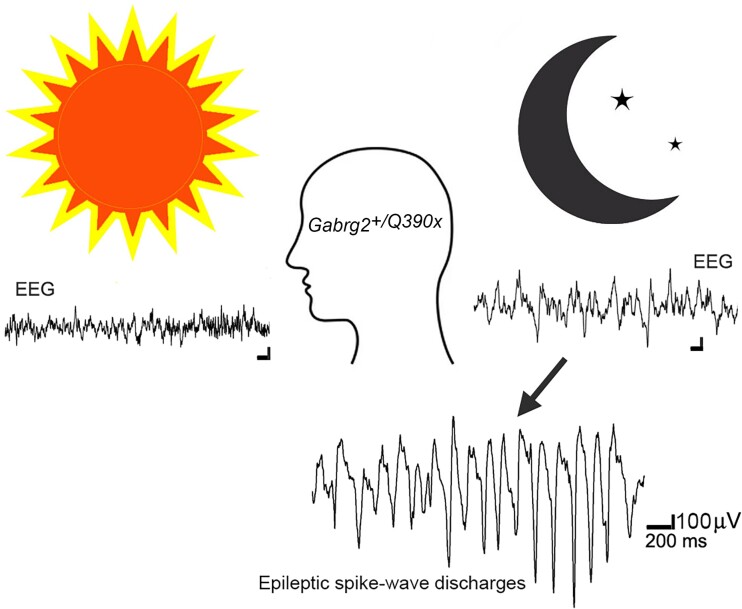

## Introduction

Sleep is the brain state when cortical activity decreases^2-5^ and the system-level memory consolidates.^6,7^ However, in human epileptic patients, including some acquired epilepsy^8^ and genetic generalized epilepsy, seizure activity exhibits preferential incidence during the sleep period.^9-16^ These nocturnal seizures can cause sleep fragmentation and decrease sleep quality in human patients,^9,15^ leading to cognitive deficits.^7,17-19^ Current cellular and network mechanisms involving thalamocortical circuitry^20-23^ can mechanistically explain ongoing seizure activity. However, there still remains lack of a clear mechanism for seizure onset/incidence modulation by day–night cycle.

Dravet syndrome is a severe genetic seizure disorder with multiple *de novo* genetic mutations identified in human patients, such as SCN1A and GABAergic receptor Gabrg2^Q390X^ mutations.^24-28^ These mutations impair the GABAergic synaptic transmission/plasticity and seizures/epileptogenesis in animal models.^26^ Moreover, Dravet syndrome patients exhibit preferential incidence of seizures during sleep, rather than wake periods.^29-31^ With mouse models containing SCN1A mutation,^25^ GABAergic interneuron dysfunction may play a major role for seizure activity generation,^25,32^ while in the het *Gabrg2^+/Q390X^* KI mouse model, GABAergic synaptic transmission and plasticity are disrupted for seizure generation.^1,33^ However, recent treatment failure with stiripentol [enhancing α3 subunit containing GABAergic receptor function^34,35^] in het *Gabrg2^+/Q390X^* KI mice^36^ suggests that there are more complicated epileptic mechanisms to explain seizure onset/treatment in the Dravet syndrome model, especially in day–night cycle modulation. Even in these Dravet animal models, there is no mechanism to address day–night cycle modulation of seizure incidence.^37^ In the same way, we still lack a mechanism for the sex difference in seizure activity in human epileptic patients,^38^ which may be linked to sex sleep/wake differences.^39-43^

Our recent work has found that artificially induced sleep-like slow-wave oscillations (SWOs) can trigger epileptic seizures in het *Gabrg2^+/Q390X^* KI mice by a sleep-related, state-dependent homeostatic synaptic potentiation mechanism.^1^ In this study, we examined if sleep SWOs themselves *in vivo* could causally trigger seizures in het *Gabrg2^+/Q390X^* KI mice, which might have implication for seizure onset mechanism and sex incidence difference in human epilepsy patients.

## Materials and methods

### Sterile mouse surgery and EEG/multi-unit recordings *in vivo*

With all procedures in accordance with the guidelines set by the institutional animal care and use committee of Vanderbilt University Medical Center and to the National Institutes of Health Guide for Care and Use of Laboratory Mice, mouse EEG surgery (C57BL6, 3–8-month-old) was performed as our previous methods and procedures that experimental mice do not suffer unnecessarily.^1^ Briefly, both wild-type (wt) littermates and het *Gabrg2^+/Q390X^* KI mice [by crossing het KI mice with NpHR expressing mice #012334 (The Jackson Laboratory)^44,45^] [both male and female mice used] underwent brain surgery [anaesthesia 1–3% isoflurane (vol/vol)] to implant three EEG screw electrodes (each for one hemisphere and one for grounding over cerebellum, Pinnacle Technology, #8201), one concentric bipolar tungsten electrode in somatosensory cortex (S1 cortex, depth 0.8∼1 mm in laminar V) and one fibre optic cannula (0.2–0.4 mm diameter, Thorlabs Inc., Newton, New Jersey) for laser light delivery *in vivo* [coordinates (within somatosensory cortex), anterior-posterior between −1.82 and −0.46 mm, midline–lateral between +2.0 and +4.0 mm reference to bregma, dorsal–ventral 0.7–1.1 mm reference to pia surface]. The tungsten electrode tip was placed slightly deeper than the optic cannula depth in S1 cortex to ensure that all neurons recorded were controlled by the laser delivered (589∼680 nm). Two EMG leads were inserted into neck trapezius muscles to monitor mouse motor activity. After surgery, mice were continuously monitored for recovery from anaesthesia and remained in the animal care facility for at least 1 week (normal sleep/wake circadian rhythm) before simultaneous EEG/EMG/multi-unit activity recordings (by persons blind to mouse genotypes). The tungsten electrode/optic cannula placement within S1 cortex was checked in mouse brains after euthanasia. All EEG (two channels, band filtered at 0.1–100 Hz), multi-unit activity (band filtered 300–2000 Hz) and EMG (band filtered at 0.1–400 Hz) were collected (all in current-clamp mode) by using two multiClamp 700B amplifiers (Molecular devices Inc., Union City, CA) and Clampex 10 software (Molecular Devices Inc., Union City, CA), and digitized at 20 kHz using a Digidata 1440 A.

The laser [DPSS laser MGL-III-589–50 (50 mW, Ultralazers Co., Inc)] was delivered through an optic fibre cable connected to the implanted optic cannula, and the laser timing was controlled by Clampex 10 software. Intracortical stimulations of 300–400 pA (20 ms) were applied to simulate neuronal up-states through the planted tungsten electrodes. SWOs *in vivo* or up–down states (0.5 Hz, for 10 min) were induced by alternating laser delivery (hyperpolarizing neurons as down-states, 1800 ms) and no laser delivery [as up-states 200 ms, with intracortical electrical stimulations (300–400 pA) at the beginning 20 ms^5,46^]. Three to four hundred pA currents and 20 ms were chosen to avoid kindling since kindling induction needs larger µA currents.^47-49^

Based on our previous study *ex vivo*,^1^ 4-(diethylamino)-benzaldehyde (DEAB) (blocking retinoid acid synthesis)^50^ was used as a drug to suppress SWO-induced homeostatic synaptic potentiation of excitatory currents in neurons *in vivo*. The DEAB was dissolved in dimethyl sulfoxide (DMSO)/saline and injected (*i.p.*) with dosages 100 mg/kg body weight, based on its pharmacokinetical potency *in vivo* to inhibit aldehyde dehydrogenase in mice.^51,52^ Injections were given once per day for 5 days [at almost the same morning time (10:00–12:00 pm) in each day] consecutively for its cumulative treatment effect. Mouse EEG recordings were performed before DEAB injections and right after the 5th injection. Control mice were injected with vehicle DMSO/saline, and no epileptic SWDs were subsequently induced. The pre-DEAB and post-DEAB EEG/EMG activity was recorded for continuous 3 h at the same circadian time of the day to avoid potential circadian variant effect on seizure incidence.

### Sleep/wake vigilance state of mouse polysomnography and epileptic SWDs identification

Both male and female mice were acclimated to recording chambers (with food/water access) for 2 days before behaviours were recorded for a 24 h period. Mouse sleep/wakeful states (24 h recordings, 12 h light/12 h dark cycle) were determined by EEG/EMG activity and video-recorded motor activity and analyzed by persons either blind to mouse genotypes or not-performing recordings. EMG activity and simultaneous video recordings were used to determine wake (continuous movement) or sleep (prolonged periods of no movement). All recorded EEG/EMG were scored as 10 s epochs of awake (low EEG amplitudes with large EMG amplitudes), non-rapid eye movement (NREM) sleep (high EEG amplitudes and dominant frequency <4 Hz), or rapid eye movement (REM) sleep (uniform/low amplitude EEG waveforms and dominant theta frequency 6–10 Hz)^6,39,53-55^ by a blinded person. For transition epochs containing both wake and sleep states, we defined a state duration >5 s as dominant. Any EEG/EMG activity artefacts associated with motor behaviours (video monitored) were removed from this analysis. EEG data will be down-sampled at 200 Hz for further Matlab (MathWorks Inc., Natick, MA) multitaper power spectral analysis.^56^ For optogenetic and DEAB experiments *in vivo*, data were obtained from EEG recordings during light phase (mouse sleep period from 10:00 am through 4:00pm) but not during 24 h recordings.

Mouse epileptic behaviours were simultaneously video-recorded with EEG recordings and Racine-scaled and analyzed by persons either blind to mouse genotypes or not-performing recordings. Bilateral synchronous SWDs (6–12 Hz) and SSWD (3–6 Hz^18,57^) were defined as trains (>1 s) of rhythmic biphasic spikes, with a voltage amplitude at least twofold higher than baseline amplitude for REM sleep and wake periods.^18,58^ During NREM sleep, due to large delta EEG amplitudes (0.5–4 Hz), bilateral synchronous SWDs (6–12 Hz, > 1 s) were defined as at least × 1.5 higher (Fig. 4 in Halasz *et al.*, 2002 and Fig. 3 and 4 in Caraballo *et al.*, 2015) than the preceding delta EEG amplitudes.^59,60^ However, these detected SWDs and SSWDs during NREM sleep or REM/wake period showed similar epileptic waveform patterns. In addition, unilateral SWD/SSWDs were defined as focal epilepsy. All atypical and typical absence epilepsy and GTCS started with SSWDs or SWDs, accompanied by characteristic motor behaviours. The behaviour associated with SWD/SSWD consisted of immobility, facial myoclonus, and vibrissal twitching. The SWD/SSWDs and animal epileptic behaviours were also checked and confirmed by persons blind to animal genotypes. Myoclonic seizures started with focal epileptic spikes in the mice, similar to A322D mice.^61^ Owing to infrequency of tonic–clonic seizures in these mice, this study did not examine this type of seizures. The onset times of SWD or SSWDs were determined by their leading-edge points crossing (either upward or downward) the precedent EEG baseline and the offset time of SWD or SSWDs by the last epileptic spikes/waves’ trailing points crossing the subsequent EEG baseline. Then the duration of SWDs/SSWDs were determined the time between the onset and offset time. Any EEG episodes with high-frequency activity and muscle artefacts associated with motor behaviours (video monitored) were removed from the power spectral analysis. With Clampfit (Molecular devices Inc., Union City, CA), NREM power spectral density of sleep EEG was averaged all 0.1–4 Hz range with continuous 30 min EEG recordings (10 s window) at the same circadian time of the recording day and portion of REM/wake EEG recordings was not used.^39^

EEG data were downsized to a 200 Hz sampling frequency for analyzing sleep spindle frequency (10–15 Hz), based on AASM manual^62^ and Kandel and Buzsaki^46^ and Holden *et al.*^63^ and using Clampfit (Molecular devices Inc., Union City, CA) and Matlab. The threshold to detect sleep spindles was set as the amplitudes that were at least 1.5 X the standard deviation larger than the preceding baseline amplitudes and whose durations were between 0.5 and 5 s.^63^

### Statistical analysis

All figures were prepared with Microsoft Excel, SigmaPlot, and Adobe Photoshop software. Power analyses were conducted with preliminary data to determine final sample sizes. Data were expressed as mean ± SEM (standard error of mean). Holm–Sidak test and one-way ANOVA were performed data in figures with SigmaStat when necessary. Otherwise, student paired/unpaired *t*-tests were used between wt and het mice or between pre- and post-groups with Excel or SigmaStat.

### Data availability

Data of original EEG/EMG recordings will be available upon reasonable request.

## Results

### Epileptic seizure incidence in het *Gabrg2^+/Q390X^* KI mice preferentially appears during NREM sleep period

**Figure 1:**
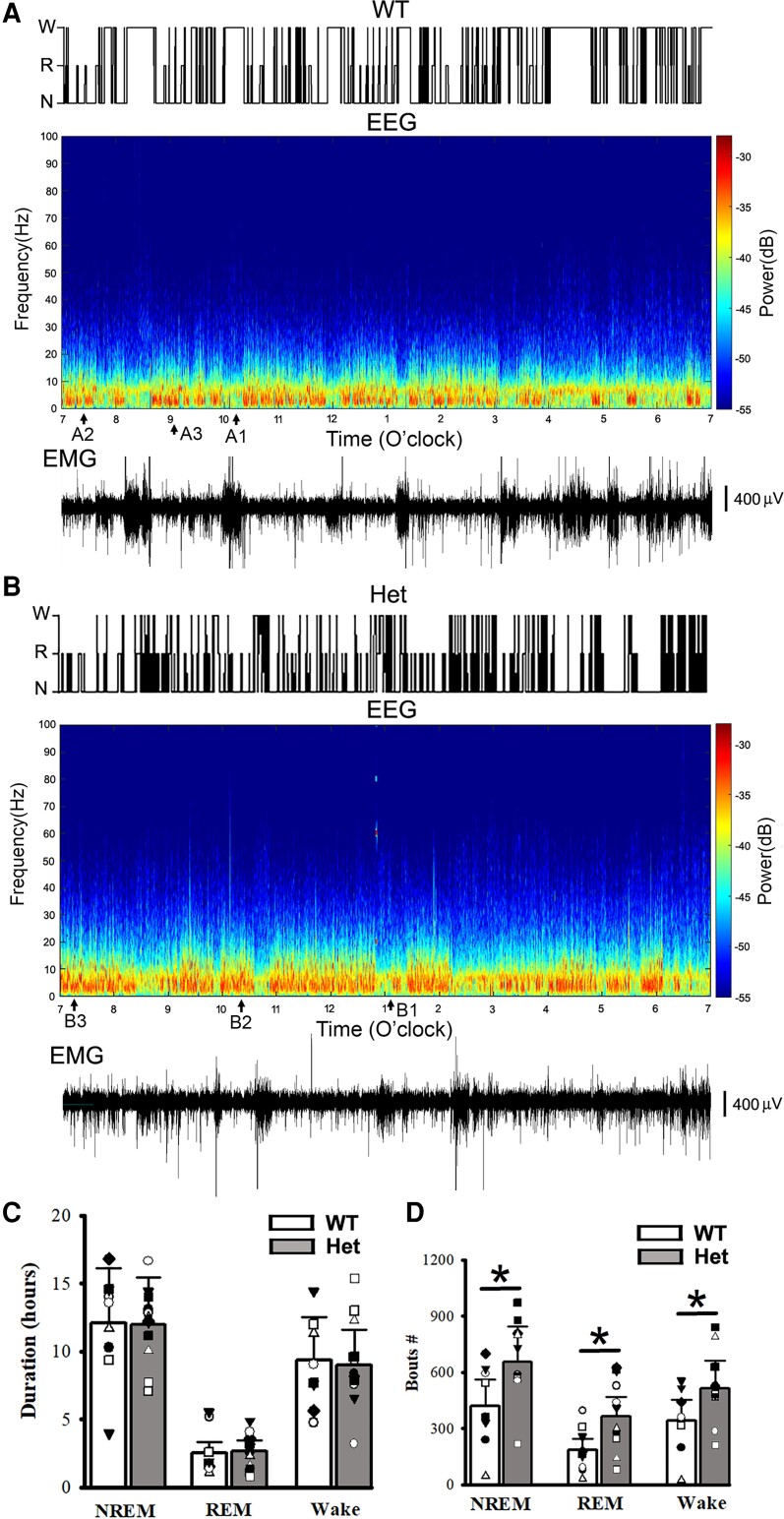
**NREM/REM sleep/wake states, power spectrograms of EEG activity and simultaneous EMG from mouse polysomnography for one wt littermate and one het *Gabrg2^+/Q390X^* KI**. Top panels in **A/B** show sleep NREM (N)/REM (R) and wake (w) states in one wt littermate and one het *Gabrg2^+/Q390X^* KI mouse from 7:00am to 7:00pm (12 h shown, from continuous 24 h recordings). Middle panels in **A/B** show the multi-tape power spectrograms of continuous EEG activity of the wt littermate and het KI mouse. Original EEG/EMG for wake, REM, and NREM sleep episodes (each 30 s long) are indicated as A1/B1, A2/B2 (very short), and A3/B3 here and are shown in Fig. 2A/B. Lower panels in A/B show simultaneous EMG activity for these EEG recordings to show mouse motor activity. Scale bars are indicated as labelled. Panels **C** shows summary duration data for NREM/REM sleep and wake period (continuous 24 h recordings) for wt *n* = 9 (male 1, female 8) and het *n* = 12 (male 6, female 6) mice. Panels **D** shows summary data of sleep NREM/REM and wake bouts. * indicates *t*-test significance with *P* < 0.05 by using *t*-test between wt and het KI (wt 9, male 1, female 8 and het 12, male 6, female 6) mice.

**Figure 2:**
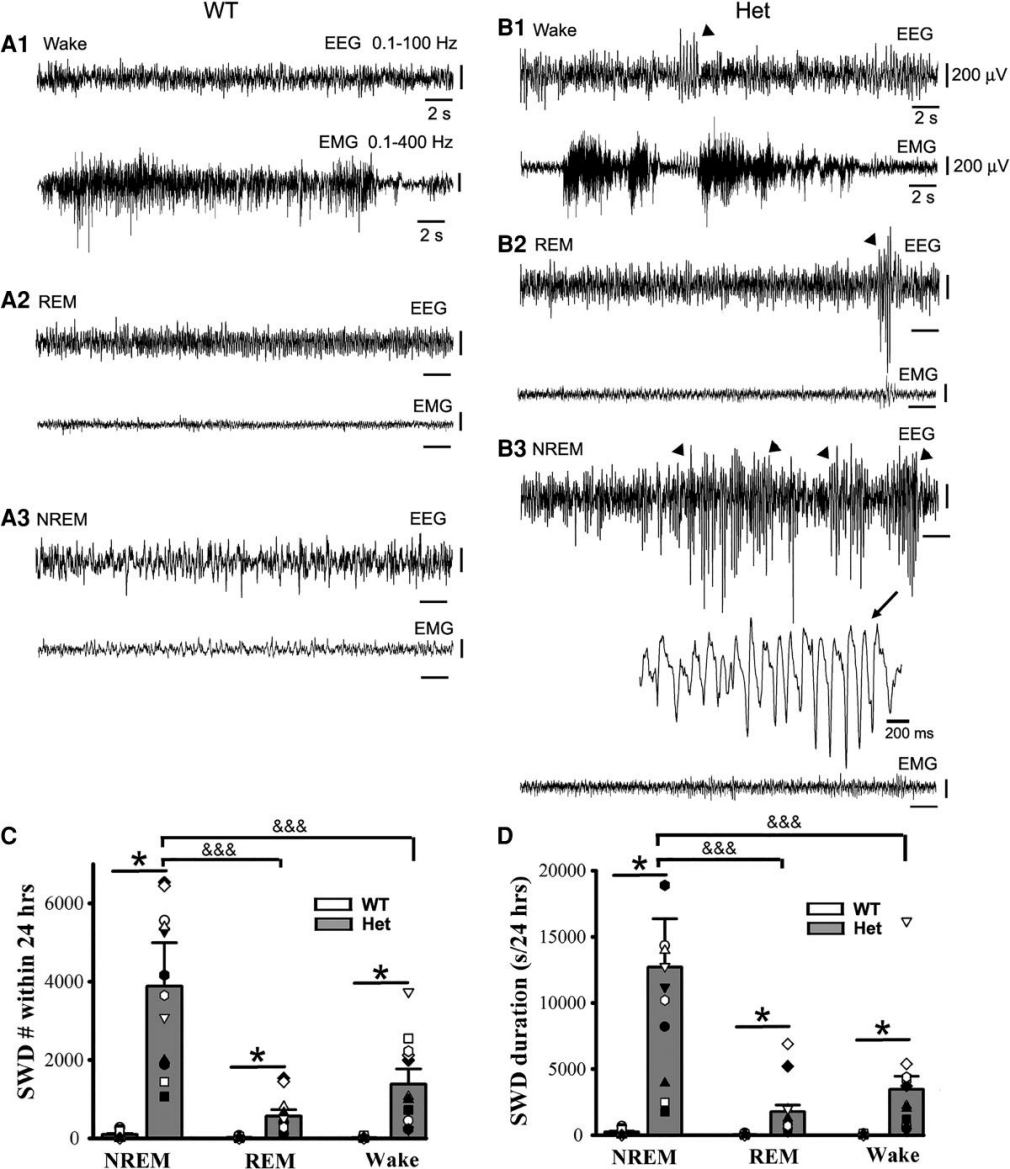
**Epileptic SWD incidence prefers NREM sleep period to REM sleep and wakeful state in het *Gabrg2^+/Q390X^* KI mice.** Panels **A/B** show representative EEG/EMG traces (30 s) for wake and REM/NREM sleep periods whose power spectrograms are indicated in Fig. 1A/B in one wt littermate and one het KI mouse. In panel B, SWDs are indicated by arrowheads and one SWD is expanded with a smaller temporal scale in panel **B3**. All scale bars are labelled as indicated. Panels **C/D** show summary data for SWD incidence and duration during NREM/REM sleep and wake periods (continuous 24 hrs recordings) for wt *n* = 9 (male 1, female 8) and het *n* = 12 (male 6, female 6) mice. * indicates significance with *P* < 0.05 by using *t*-test between wt and het in NREM or REM or wake groups and &&& one-way ANOVA significance with *P* < 0.05 between NREM, REM and wake groups in het mice (also see result section).

Using 24 h continuous recordings after mouse habituation, mouse active or rest states, EEG patterns and epileptic SWD activity during sleep and wakeful period were examined from wt littermates and het *Gabrg2^+/Q390X^* KI mice. As indicated in Fig. 1 and 2, EEG activity during NREM sleep period exhibited large amplitudes with chaotic waveforms (Fig. 2 A3/B3) and dominant delta EEG frequency (Fig. 1), while there was no motor activity (EMG within Fig. 2 A3/B3). In contrast, EEG activity during REM sleep showed more uniform waveforms with small amplitudes and dominant theta frequency 6–10 Hz (Fig. 1 and 2 A2/B2). Also EEG activity during wakeful period exhibited much smaller amplitude waveforms and higher frequencies (from theta to gamma ranges) (Fig. 1 and 2 A1/B1). Moreover, we did not find any significant differences in NREM/REM/wake duration between wt and het *Gabrg2^+/Q390X^* KI mice [hours (hrs) NREM: wt (*n* = 9, male 1, female 8) 12.09 ± 4.03 versus het (*n* = 12, male 6, female 6) 12.01 ± 3.46, *t*-test *P* = 0.94; REM wt 2.52 ± 0.84 versus het 2.67 ± 0.77, *t*-test *P* = 0.813; wake wt 9.39 ± 3.13 versus het 8.99 ± 2.60, t-tests *P* = 0.783]. In addition, compared with wt littermates, sleep in het *Gabrg2^+/Q390X^* KI mice seemed to be more fragmented (Fig. 1D) with more NREM/REM/wake bouts (NREM #: wt (*n* = 9, male 1, female 8) 421.67 ± 140.56 versus het (*n* = 12, male 6, female 6) 655.00 ± 189.08, *t*-test *P* = 0.017; REM wt 184.11 ± 61.37 versus het 363.58 ± 104.95, *t*-test *P* = 0.029; wake wt 340.67 ± 113.56 versus het 515.25 ± 148.73, t-tests *P* = 0.03) and a trend of increased sleep-state transition [NREM >> wake transition: wt (*n* = 9) 259.11 ± 86.37 versus het (*n* = 12) 399.17 ± 115.23, *t*-test *P* = 0.081; REM >> wake transition: wt (*n* = 9) 81.33 ± 27.11 versus het (*n* = 12) 115.75 ± 33.41, *t*-test *P* = 0.262].

Unlike EEG patterns in wt littermates, EEG activity in het *Gabrg2^+/Q390X^* KI mice exhibited significantly more epileptic SWDs (Fig. 2 B1–3 and C/D) [24 h recordings, SWD# during NREM: wt (*n* = 9, male 1, female 8) 95.11 ± 31.70 versus het (*n* = 12, male 6, female 6) 3876 ± 1119.5, *t*-test *P* = 0.0001; during REM wt 17.22 ± 5.74 versus het 566 ± 163.39, *t*-test *P* = 0.003; during wakeful period wt 15.33 ± 5.1 versus het 1378.16 ± 397.84, *t*-test *P* = 0.002] [24 h recordings, SWD duration(s) during NREM: wt (*n* = 9, male 1, female 8) 237.13 ± 79.04 (0.54 ± 0.18% of total NREM duration) versus het (*n* = 12, male 6, female 6) 12 698.36 ± 3665.70 (28.09 ± 8.10% of total NREM duration), *t*-test *P* = 0.0006; during REM wt 39.25 ± 13.08 (0.43 ± 0.14% of total REM duration) versus het 1769.91 ± 510.93 (17.86 ± 5.15% of total REM duration), *t*-test *P* = 0.024; during wakeful period wt 31.63 ± 10.54 (0.13 ± 0.04% of total wake) versus het 3455.65 ± 997.56 (11.56 ± 3.34% of total wake), *t*-test *P* = 0.031]. Particularly, epileptic SWD incidence preferentially occurred during NREM sleep period (Fig. 2 C/D) [het (*n* = 12, male 6, female 6) SWD# during NREM/REM/wake, one-way ANOVA *P* = 0.005 and NREM versus REM *P* = 0.017, NREM versus wake *P* = 0.025; SWD duration during NREM/REM/wake, one-way ANOVA *P* < 0.001 and NREM versus REM *P* = 0.00003, NREM versus wake *P* = 0.0003]. These results indicate that in this genetic epilepsy het *Gabrg2^+/Q390X^* KI mice, epileptic SWD preferential incidence during NREM sleep period is very similar to sleep preferential incidence of epileptic activity in human Dravet syndrome patients.

### Epileptic SWD incidence in female het *Gabrg2^+/Q390X^* KI mice is more regulated by NREM sleep SWOs *in vivo*

**Figure 3:**
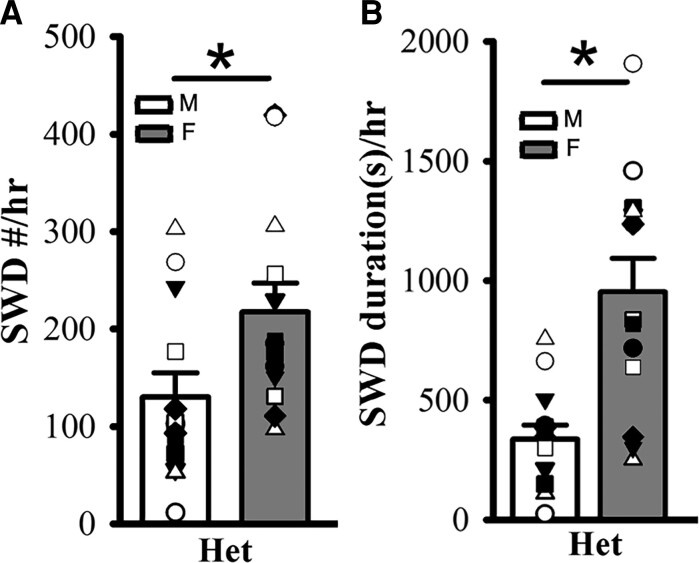
**Female het *Gabrg2^+/Q390X^* KI mice exhibit more seizure incidence than male het *Gabrg2^+/Q390X^* KI mice.** Panels **A/B** show summary SWD incidence and duration data from male (M, blank) and female (F, filled) het *Gabrg2^+/Q390X^* KI mice, with male and female het KI mice (*n* = 13 each). * indicates *t*-test significance with *P* < 0.05.

In addition, we found significantly more SWD incidence and longer SWD duration in female het *Gabrg2^+/Q390X^* KI mice compared with male het *Gabrg2^+/Q390X^* KI mice (Fig. 3A/B) [SWD #/hr, male (*n* = 13), 129.98 ± 24.98 versus female 217.58 ± 29.41 (*n* = 13), *t*-test *P* = 0.032; SWD duration(s)/h, male 336.81 ± 59.59 versus female 952.03 ± 140.00, *t*-test *P* = 0.0005].

### Induced SWOs *in vivo* can trigger epileptic SWD incidence in het *Gabrg2^+/Q390X^* KI mice

**Figure 4:**
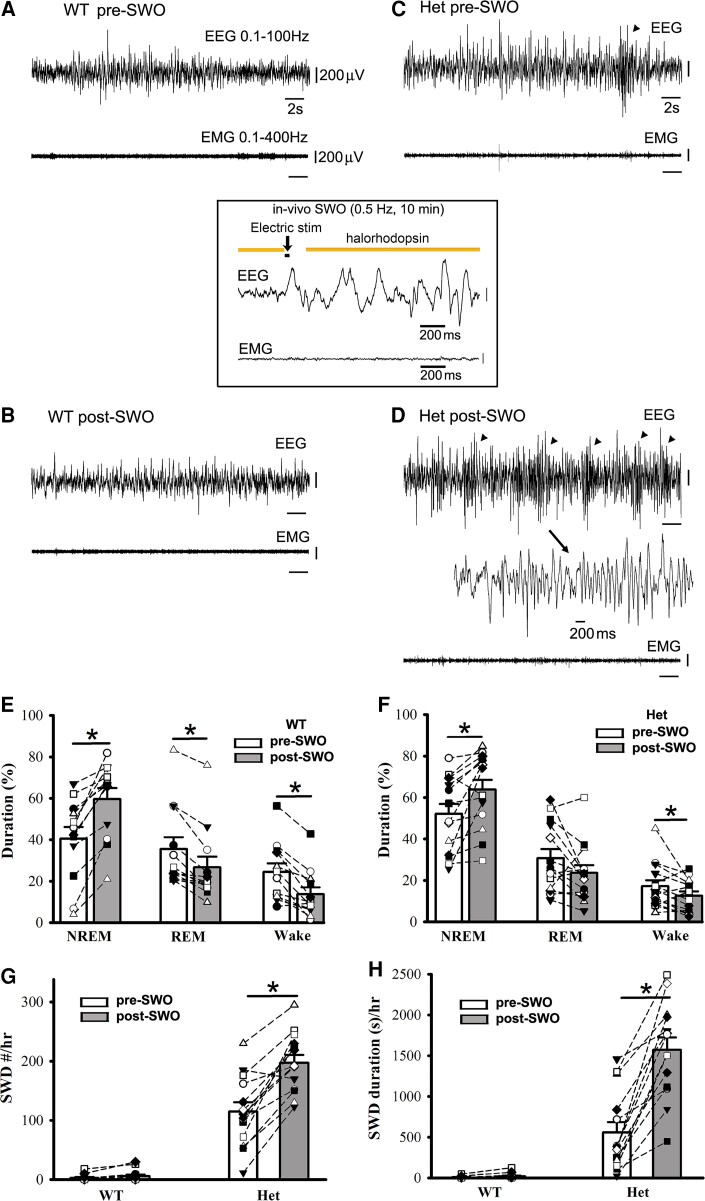
**Optogenetically induced SWOs *in vivo* increase NREM sleep duration and trigger more epileptic SWDs in het *Gabrg2^+/Q390X^* KI mice.** Panels **A/B** show representative pre-SWO and post-SWO EEG/EMG traces (30 s) in one wt littermate. Panels **C/D** show representative pre-SWO and post-SWO EEG/EMG traces (30 s) in one het KI mouse. Inset is one representative SWO *in vivo* (2 s episode, total 10 min) with optogenetic activation of NpHR channels. SWDs are indicated as arrowheads and one SWD is expanded with a smaller temporal scale in panel **D**. All scale bars are labelled as indicated. Panels **E/F** show summary duration data of pre-SWO and post-SWO NREM/REM/wake period from wt littermates (*n* = 12, male 6, female 6) and het KI (*n* = 14, male 7, female 7) mice (total NREM/REM/wake duration are converted as 100% for prior SWO or post-SWO period). Panels **G/H** show summary SWD incidence data for wt littermates (*n* = 14, male 7, female 7) and het KI mice (*n* = 14, male 7, female 7). * indicates paired *t*-test significance with *P* < 0.05 between pre-SWO and post-SWO.

EEG activity during NREM sleep exhibits slow-wave oscillations within delta-frequency range accompanied by simultaneous neuronal up- and down-state activity in the cerebral cortex. Thus, we examined whether induction of slow-wave oscillation (SWOs, 0.5 Hz) *in vivo* could change NREM sleep duration and trigger epileptic SWDs in het *Gabrg2^+/Q390X^* KI mice. With mice expressing NpHR proteins in cortical neurons, we optogenetically induced SWOs by hyperpolarizing neurons with 593 yellow laser light *in vivo* [Zhang *et al.*^1^ ] and intracortical stimulation (400 pA, 20 ms) (Fig. 4 inset). When given 0.5 Hz SWOs induction for 10 min (total NREM/REM sleep/wake duration were converted to 100% during pre/post-SWO observation period), NREM sleep duration was significantly increased for both wt and het *Gabrg2^+/Q390X^* KI mice (Fig. 4E/F) [wt (*n* = 12, male 6, female 6) pre-SWO 40.40 ± 5.78% versus post-SWO 59.60 ± 5.33%, paired *t*-test *P* = 0.00001; het (*n* = 14, male 7, female 7) pre-SWO 52.04 ± 4.89% versus post-SWO 63.77 ± 4.76%, paired *t*-test *P* = 0.007]. However, under the same conditions, REM sleep and wake duration were slightly decreased in wt and het *Gabrg2^+/Q390X^* KI mice (except REM sleep) (Fig. 4E/F) [REM sleep, wt (*n* = 12, male 6, female 6) pre-SWO 35.53 ± 5.71% versus post-SWO 26.62 ± 5.29%, paired *t*-test *P* = 0.0003; het (*n* = 14, male 7, female 7) pre-SWO 30.76 ± 4.35% versus post-SWO 23.64 ± 3.67%, paired *t*-test *P* = 0.075] [wake, wt (*n* = 12, male 6, female 6) pre-SWO 24.43 ± 4.10% versus post-SWO 13.69 ± 3.34%, paired *t*-test *P* = 0.0003; het (*n* = 14, male 7, female 7) pre-SWO 17.18 ± 2.92% versus post-SWO 12.58 ± 2.09%, paired *t*-test *P* = 0.0492]. Correspondingly, following *in vivo* SWO induction, epileptic SWDs in het *Gabrg2^+/Q390X^* KI mice, but not wt littermates, significantly increased in both incidence number and duration (even reached status epilepticus in some het KI mice)(Fig. 4C/D/G/H) which were also accompanied by mouse immobility or minor facial muscle twitching (Fig. 4A–D EMG) [SWD #/hr, wt (*n* = 14, male 7, female 7) pre-SWO 2.78 ± 1.38 versus post-SWO 5.71 ± 2.64, paired *t*-test *P* = 0.08; het (*n* = 14, male 7, female 7) pre-SWO 114.86 ± 15.70 versus post-SWO 197.38 ± 13.31, paired *t*-test *P* = 0.00001] [SWD duration(s)/hr, wt (*n* = 14, male 7, female 7) pre-SWO 6.72 ± 3.68 versus post-SWO 18.81 ± 9.61, paired *t*-test *P* = 0.073; het (*n* = 14, male 7, female 7) pre-SWO 557.96 ± 128.33 versus post-SWO 1571.28 ± 153.76, paired *t*-test *P* = 0.000004]. These findings likely indicate that SWO induction *in vivo* can increase NREM sleep duration in all mice and trigger epileptic SWDs in het *Gabrg2^+/Q390X^* KI mice.

### Suppression of synaptic potentiation *in vivo* drastically decreases epileptic SWDs in het *Gabrg2^+/Q390X^* KI mice

**Figure 5:**
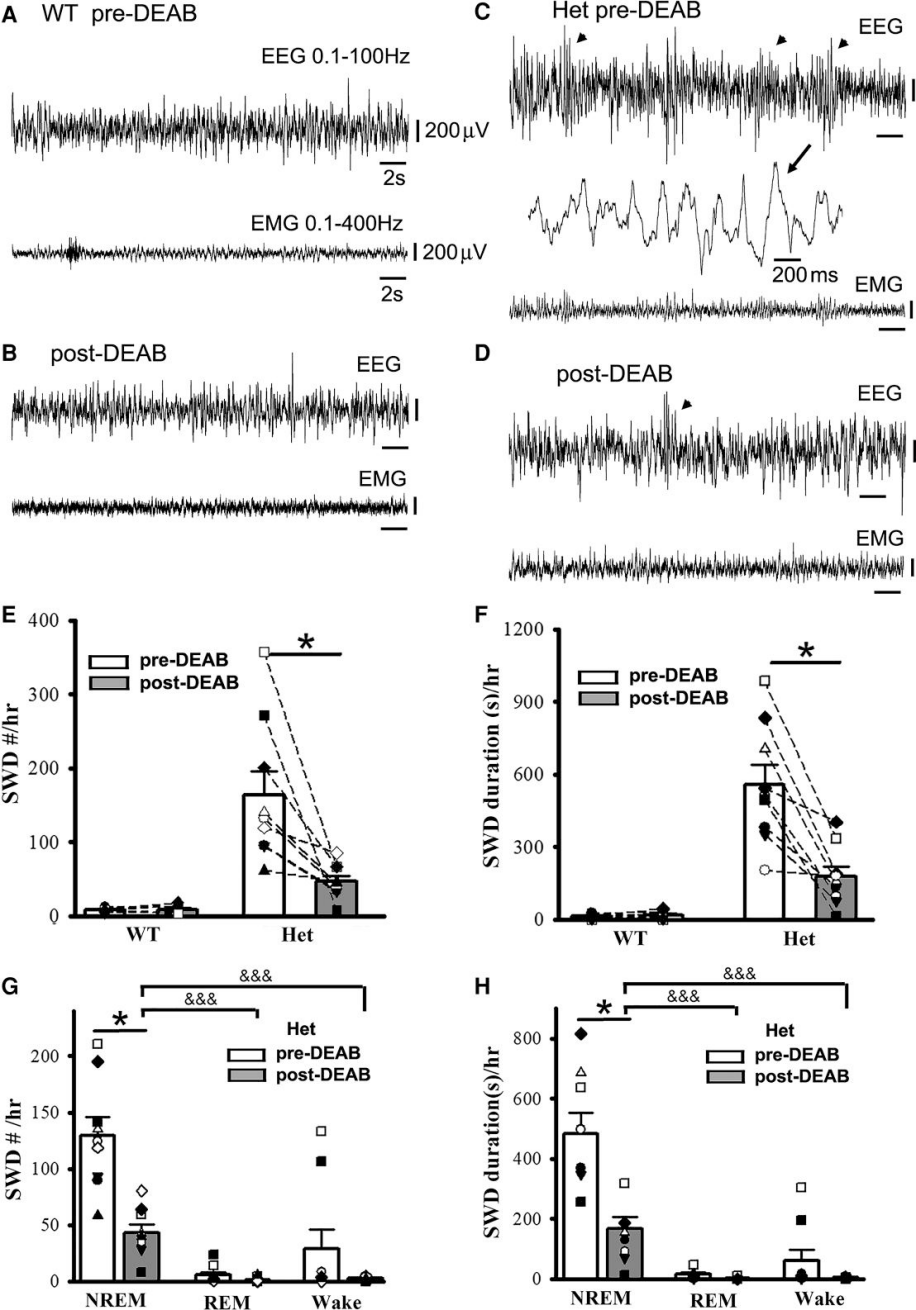
**Suppression of SWO-induced homeostatic synaptic potentiation by DEAB decreases epileptic SWD incidence in het *Gabrg2^+/Q390X^* KI mice.** Panels **A/B** show representative pre-DEAB and post-DEAB EEG/EMG traces (30 s) in one wt littermate. Panels **C/D** show representative pre-DEAB and post-DEAB EEG/EMG traces (30 s) in one het KI mouse. SWDs are indicated as arrowheads, and one SWD is expanded with a smaller temporal scale in panel **C**. All scale bars are labelled as indicated. Panels **E/F** show summary SWD incidence and duration data for wt littermates (*n* = 7, male 5, female 2) and het KI mice (*n* = 9, male 2, female 7). Panels **G/H** show summary pre-DEAB and post-DEAB SWD incidence and duration data from het KI mice during NREM/REM/wake period. * indicates paired *t*-test significance with *P* < 0.05 between pre-DEAB and post-DEAB in panels **E–H** (also see result section). One-way ANOVA was tested for pre-DEAB (not shown) or post-DEAB (shown) NREM, REM and wake period for panels **G** and **H**, with significance &&& *P* < 0.00001.

It is difficult to find a way to suppress SWOs *in vivo* without influencing cortical neuronal excitability and mouse sleep states. Our previous work^1^ and other works^64^ have indicated that SWOs induce homeostatic synaptic potentiation in cortical neurons, which depends on retinoid acid synthesis of the enzyme aldehyde dehydrogenase. Thus, DEAB (100 mg/kg weight)^51^ was used to block aldehyde dehydrogenase as an alternative method to suppress SWO-related homeostatic synaptic potentiation *in vivo*.^50^ With consecutive five-day DEAB injections (*i.p.*), we did not find any significant changes in NREM/REM/wake duration from wt and het *Gabrg2^+/Q390X^* KI mice [NREM duration(s)/3 h at the same circadian time of days, wt (*n* = 7, male 5, female 2) pre-DEAB 6864.28 ± 234.74 versus post-DEAB 6788.55 ± 277.34, paired *t*-test *P* = 0.86; het (*n* = 9, male 2, female 7) pre-DEAB 7506.67 ± 663.45 versus post-DEAB 7238.89 ± 411.63, paired *t*-test *P* = 0.751] [REM duration(s)/3 hrs, wt (*n* = 7, male 5, female 2) pre-DEAB 353.43 ± 71.35 versus post-DEAB 271.59 ± 48.68, paired *t*-test *P* = 0.391; het (*n* = 9, male 2, female 7) pre-DEAB 394.44 ± 48.73 versus post-DEAB 276.67 ± 62.38, paired *t*-test *P* = 0.167] [Wake duration(s)/3 hrs, wt (*n* = 7, male 5, female 2) pre-DEAB 3582.28 ± 243.83 versus post-DEAB 3738.40 ± 262.89, paired *t*-test *P* = 0.734; het (*n* = 9, male 2, female 7) pre-DEAB 2898.89 ± 636.17 versus post-DEAB 3284.44 ± 355.78, paired *t*-test *P* = 0.626]. However, following DEAB injections *in vivo*, epileptic SWDs in het *Gabrg2^+/Q390X^* KI mice significantly decreased in incident number and duration (Fig. 5E/F), while in wt littermates there were no epileptic SWDs *in vivo* triggered (Fig. 5E/F) [SWD #/h, wt (*n* = 7, male 5, female 2) pre-DEAB 7.85 ± 0.86 versus post-DEAB 8.54 ± 2.30, paired *t*-test *P* = 0.751; het (*n* = 9, male 2, female 7) pre-DEAB 163.85 ± 32.05 versus post-DEAB 47.03 ± 7.63, paired *t*-test *P* = 0.007] [SWD duration (s)/h, wt (*n* = 7, male 5, female 2) pre-DEAB 13.58 ± 1.94 versus post-DEAB 17.88 ± 5.79, paired *t*-test *P* = 0.413; het (*n* = 9, male 2, female 7) pre-DEAB 556.87 ± 82.81 versus post-DEAB 176.80 ± 41.06, paired *t*-test *P* = 0.0009]. More interestingly, epileptic SWDs in het *Gabrg2^+/Q390X^* KI mice were significantly suppressed during NREM sleep but not REM sleep and wake period (Fig. 5G/H). [NREM SWD #/hr, het (*n* = 9, male 2, female 7) pre-DEAB 129.59 ± 16.35 versus post-DEAB 43.63 ± 7.67, paired *t*-test *P* = 0.0003] [REM SWD #/hr, het (*n* = 9, male 2, female 7) pre-DEAB 5.63 ± 2.64 versus post-DEAB 1.41 ± 0.63, paired *t*-test *P* = 0.16] [wake SWD #/h, het (*n* = 9, male 2, female 7) pre-DEAB 28.59 ± 17.40 versus post-DEAB 2.48 ± 0.57, paired *t*-test *P* = 0.18] [NREM SWD duration (s)/h, het (*n* = 9, male 2, female 7) pre-DEAB 482.39 ± 69.22 versus post-DEAB 167.61 ± 39.36, paired *t*-test *P* = 0.0009] [REM SWD duration (s)/h, het (*n* = 9, male 2, female 7) pre-DEAB 15.03 ± 6.17 versus post-DEAB 4.08 ± 1.89, paired *t*-test *P* = 0.11] [wake SWD #/h, het (*n* = 9, male 2, female 7) pre-DEAB 59.41 ± 37.09 versus post-DEAB 5.11 ± 1.56, paired *t*-test *P* = 0.19]. In addition, both pre-DEAB and post-DEAB SWD incidence preferred NREM sleep period (both one-way ANOVA *P* < 0.00001). Together with the results of SWO induction *in vivo*, these results indicate that SWOs *in vivo* do causally trigger epileptic SWDs in het *Gabrg2^+/Q390X^* KI mice and SWOs may create brain states to trigger or facilitate epileptic SWD generation in het KI mice. However, SWOs seem not to influence myoclonic jerk generation in het *Gabrg2^+/Q390X^* KI mice.

### Sleep spindles are also regulated by SWO induction *in vivo* and DEAB administration

**Figure 6:**
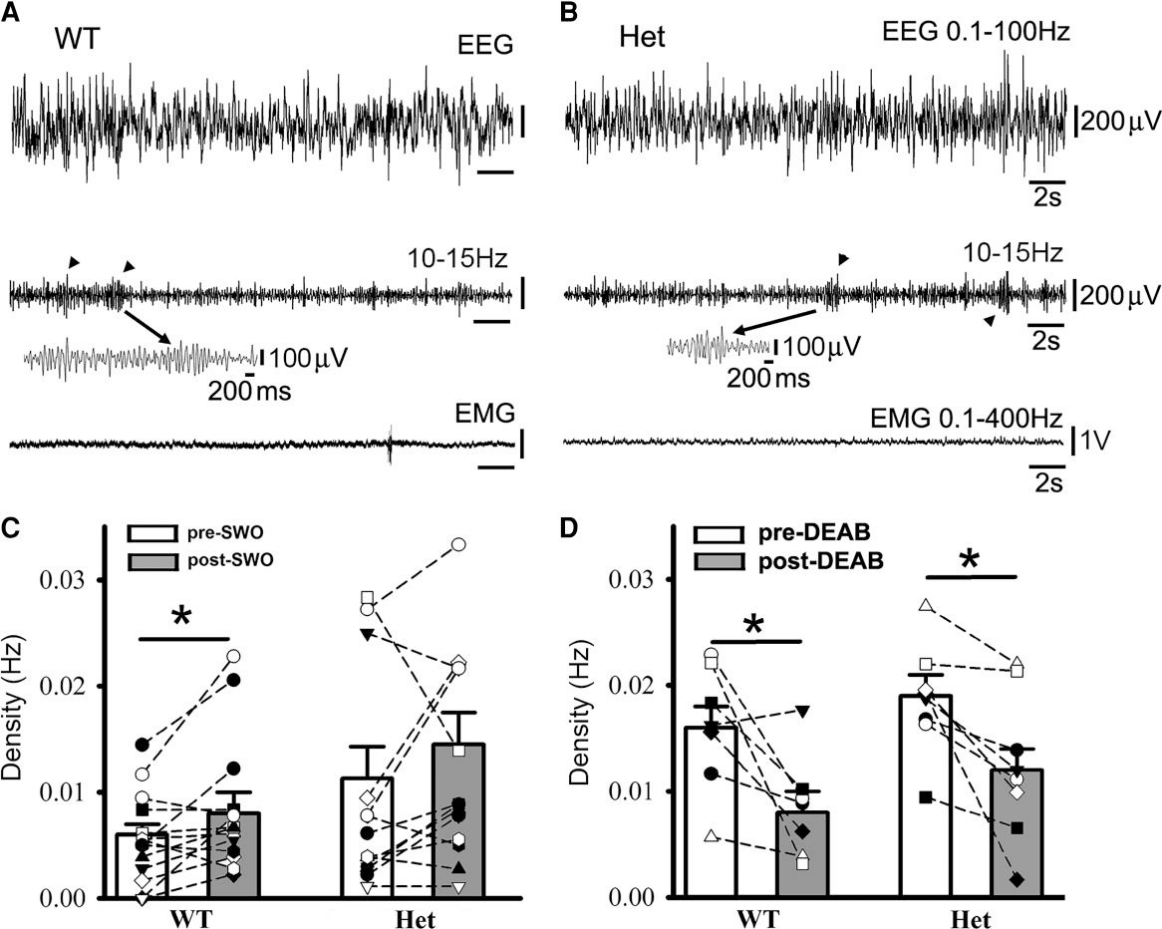
**Sleep spindles in wt littermates and het *Gabrg2^+/Q390X^* KI mice following SWO induction *in vivo* or DEAB injection *i.p.*** Panels **A/B** show representative pre-DEAB original simultaneous EEG/EMG traces (30 s long) during NREM sleep period and sleep spindles (10–15 Hz) detected (arrowheads in the middle panels) for one wt littermate (**A**) and one het KI mouse (**B**). Individual spindle events below are expanded in smaller temporal scales. Panels **C/D** show summary data for sleep spindles following SWO induction *in vivo* (panel **C**, wt *n* = 14, male 7, female 7; het *n* = 15, male 7, female 8 mice) or DEAB injection (panel **D**, wt *n* = 7, male 5, female 2; het *n* = 8, male 2, female 6 mice). All scale bars are labelled as indicated. * indicates paired *t*-test significance with *P* < 0.05.

It has been shown that with the same thalamocortical circuitry, sleep spindles can evolve into epileptic SWDs under pathological circumstances.^20,21^ Thus, we reason that SWO induction and DEAB administration *in vivo* would regulate the sleep spindle generation in wt littermates and het *Gabrg2^+/Q390X^* KI mice (Fig. 6A/B expanded events). When SWOs were optogenetically induced, sleep spindles were increased in wt littermates but not in het *Gabrg2^+/Q390X^* KI mice (Fig. 6C) [density (Hz), wt pre-SWO 0.0056 ± 0.0011 versus post-SWO 0.0084 ± 0.0016, paired *t*-test *P* = 0.022, *n* = 14, male 7, female 7; het pre-SWO 0.0113 ± 0.0031 versus post-SWO 0.0145 ± 0.0033, paired *t*-test *P* = 0.102; *n* = 15, male 7, female 8]. These results suggest that homeostatic synaptic potentiation involved in sleep spindle generation and seizure activity has already hijacked and occluded spindle generation in het *Gabrg2^+/Q390X^* KI mice. Moreover, DEAB administration *in vivo* suppressed sleep spindles in both wt littermates and het *Gabrg2^+/Q390X^* KI mice (Fig. 6D) [density (Hz), wt, pre-DEAB 0.0161 ± 0.0023 versus post-DEAB 0.0085 ± 0.0018, paired *t*-test *P* = 0.031; *n* = 7, male 5, female 2; het, pre-DEAB 0.0186 ± 0.0018 versus post-DEAB 0.0123 ± 0.0024, paired *t*-test *P* = 0.011; *n* = 8, male 2, female 6]. Moreover, DEAB administration *in vivo* suppressed more spindles in wt littermate than het KI mice, indicating that homeostatic synaptic potentiation contributed to the sleep spindle generation in a similar way as epileptic SWDs. However, sleep spindle duration seemed not to be influenced by SWO induction or DEAB administration *in vivo*.

### Female het *Gabrg2^+/Q390X^* KI mice exhibit larger delta-frequency power during NREM sleep than Male het *Gabrg2^+/Q390X^* KI mice

**Figure 7:**
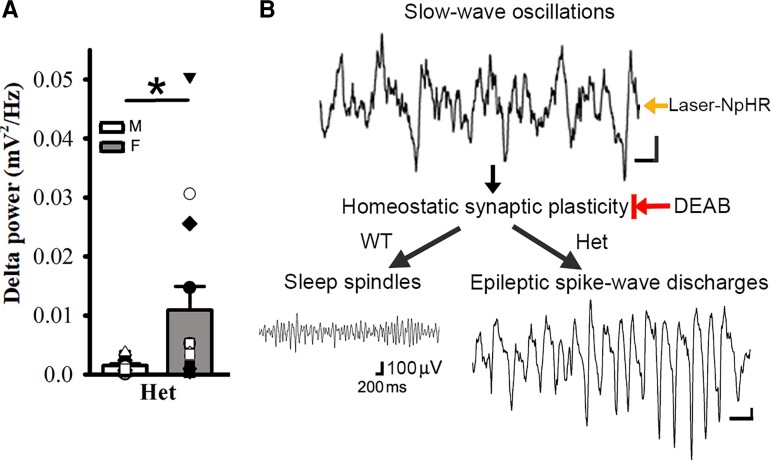
**Female het *Gabrg2^+/Q390X^* KI mice exhibit larger delta-frequency power during NREM sleep than male het *Gabrg2^+/Q390X^* KI mice and the diagram for seizure onset in het *Gabrg2^+/Q390X^* KI mice.** Panels **A** shows summary data of the power of delta-frequency (0.1–4 Hz) EEG activity during NREM sleep period for male (M, blank) and female (F, filled) het KI mice (*n* = 13 each). * indicates *t*-test significance with *P* < 0.05. Panels **B** shows the mechanism/pathway diagram in the study. All scale bars are labelled as with the same scale indicated. The black arrows in the diagram indicate the sequential pathways for epileptic SWD onset. The Laser-NpHR arrow indicates the laser-activated NpHR to generate SWOs *in vivo*, and the DEAB arrow with a vertical bar indicates the DEAB suppression of homeostatic synaptic plasticity *in vivo*.

Since SWO induction *in vivo* in het *Gabrg2^+/Q390X^* KI mice could trigger epileptic SWDs, we reasoned that without any significant difference in NREM/REM/wake duration between het female and male KI *Gabrg2^+/Q390X^* mice, delta-power of NREM sleep in female and male mice might contribute the gender difference in epileptic SWD incidence. As expected, female het *Gabrg2^+/Q390X^* KI mice did show a larger amplitude power within EEG delta frequency (0.1–4 Hz) than male het *Gabrg2^+/Q390X^* KI mice (Fig. 7A) [mV^2^/Hz, male (*n* = 13), 0.0014 ± 0.00034 versus female 0.0109 ± 0.0042 (*n* = 13), *t*-test *P* = 0.037], suggesting that NREM sleep difference in delta-frequency power may generate the gender difference in epileptic SWD incidence during sleep in human Dravet syndrome patients.

## Discussion

In this study, we have found that seizure incidence in het *Gabrg2^+/Q390X^* KI mice exhibited NREM sleep preference, as compared with REM sleep or wake periods. Furthermore, artificially induced SWOs *in vivo* (by optogenetic method) could significantly trigger more epileptic SWDs in het *Gabrg2^+/Q390X^* KI mice, while *in vivo* suppression of SWO-related homeostatic synaptic potentiation by DEAB injections significantly decreased the SWD incidence in het *Gabrg2^+/Q390X^* KI mice (Fig. 7B diagram). Meanwhile, SWO induction or DEAB injection could regulate sleep spindle generation in wt and SWDs in het *Gabrg2^+/Q390X^* KI mice. Together, these results suggest that sleep SWOs themselves *in vivo* could trigger epileptic SWD onset. In addition, in female het *Gabrg2^+/Q390X^* KI mice, EEG delta-frequency (0.1–4 Hz) power during NREM sleep was significantly larger than that in male het *Gabrg2^+/Q390X^* KI mice, which likely contributes to gender difference in seizure incidence in human Dravet syndrome patients.

During sleep periods, cortical neurons in the brain exhibit a firing activity decrease (also called as cortical disfacilitation^65,66^) compared with their firing activity during wake period.^3,5^ Moreover, during sleep, neurons undergo up-down activity state alterations^5,67,68^ and balanced excitatory and inhibitory synaptic plasticity in cortical neurons is actively engaged for memory consolidation,^69-71^ while the whole-brain EEG activity simultaneously exhibits delta-frequency range SWOs.^72,73^ In brain injury models, the cortical disfacilitation has been invoked for epileptogenesis.^8,74,75^ Our previous work has shown that NREM sleep presents itself as a special brain state with impaired homeostatic synaptic potentiation of inhibitory currents in cortical neurons in het *Gabrg2^+/Q390X^* KI mice^1^ and NREM sleep creates an unbalanced/run-away excitatory synaptic potentiation in cortical neurons by SWOs. This brain state will trigger the seizure onset in het *Gabrg2^+/Q390X^* KI mice during NREM sleep/quiet–wakeful state. Consistent with this mechanism, the current work here indicates that epileptic seizure incidence does show preferential modulation by NREM sleep in het *Gabrg2^+/Q390X^* KI mice. Moreover, artificially induced SWOs *in vivo* or suppressing SWO-related homeostatic excitatory synaptic potentiation in cortical neurons correspondingly enhances or decreases epileptic SWD incidence and duration respectively, suggesting that SWOs themselves *in vivo* causally trigger epileptic seizure onset. This seizure onset scenario is the first mechanistically established in epilepsy models and neuroscience fields as we know. Particularly, induced SWOs *in vivo* can increase SWD duration up to status epilepticus in some het *Gabrg2^+/Q390X^* KI mice. Moreover, in SCN1A Dravet model,^25,26^ interneuron dysfunction in the cortex^32^ may cause the similar seizure onset mechanisms as het *Gabrg2^+/Q390X^* KI mice, given that in SCN1A animal models, only GABAergic synaptic transmission/plasticity is impaired.^76-78^ However, for Dravet models with other mutations than GABAergic transmission or receptors,^26,79^ more work are required to explore their seizure onset mechanism, particularly during sleep period. Consistent with this SWO-trigger seizure mechanism, female het *Gabrg2^+/Q390X^* KI mice show larger spectral power of delta-frequency range than male het *Gabrg2^+/Q390X^* KI mice, which is compatible with sex-dependent difference in sleep delta activity^40-43^ and very likely contributes to more seizure incidence in female het *Gabrg2^+/Q390X^* KI mice than male het *Gabrg2^+/Q390X^* KI mice. This explains in Dravet syndrome patients why female have more seizures than male.^27,29,31,80^

Since this SWO-triggered seizure mechanism only generates the brain state for seizure onset, it does not create epileptic neuron engrams *de novo* in the brain for het *Gabrg2^+/Q390X^* KI mice. This is compatible with the finding that DEAB injections (*i.p.*) [based on 100 mg/kg weight dosage from literatures^51,52^] only suppresses 70% of seizure incidence in het *Gabrg2^+/Q390X^* KI mice. Higher DEAB dosage administration may be needed for the therapeutic treatment with its maximal potency *in vivo*. However, this SWO-related mechanism might determine the seizure duration for status epilepticus evolution (see Fig. 4H). Due to the temporal overlap of SWO-triggered seizure mechanism with NREM sleep memory consolidation,^7^ this seizure onset mechanism in het *Gabrg2^+/Q390X^* KI mice can hijack/occlude sleep spindle generation (Fig. 6C/D) in the cortex circuitry for memory consolidation to impair memory formation and eventually cognitive deficit may develop in human Dravet syndrome patients.^19,25,81^ Moreover, this mechanism may cause chronic development of cognitive deficits due to childhood absence epilepsy which shows seizure activity modulation by sleep–wake cycles.^8,15,18,19^ Moreover, DEAB suppression of the majority seizure incidence may promote novel treatments of drug-resistance seizures, considering that Dravet syndrome patients have drug-resistant seizures,^26^ and 30% epileptic patients are not seizure-free after conventional treatments.^12,82-86^

However, due to half-life time of DEAB^51,52^ in mice/rats and unknown peripheral actions of DEAB on other aldehyde dehydrogenases^87,88^ in mice, we do not know the exact intracerebral DEAB concentration in the brain following *i.p.* injection of DEAB (100 mg/kg body weight) and its subsequent effects on peripheral organs such as liver and heart (mitochondria),^89,90^ which can cause new seizure onset or exacerbate seizures in human patients with aldehyde hydrogenase mutations^91,92^ (comparable with acute DEAB action *in vivo*). Thus, we cannot exclude potential seizure-prone nonessential effects of DEAB in peripheral organs (if this happens), which might cause varied DEAB suppressing effects on epileptic activity in different het *Gabrg2^+/Q390X^* KI mice and is the limit to interpret the result of DEAB treatment *in vivo* in this study. Overall, our data show that the blocking aldehyde hydrogenase with DEAB *in vivo* (DEAB can be lipid-dissolved^51^) suppresses epileptic activity, indicating that the homeostatic synaptic plasticity does contribute to seizure incidence/onset mechanism in the het *Gabrg2^+/Q390X^* KI mouse model.

All together this study suggests a novel mechanism of SWO-triggered seizures in het *Gabrg2^+/Q390X^* KI mice for one genetic generalized epilepsy such as Dravet syndrome due to *Gabrg2^Q390X^* mutation. This mechanism may contribute to chronic development of status epilepticus and likely determines the gender difference of seizure incidence and leads to the cognitive deficits in human genetic epilepsy patients.

## Abbreviations

DEAB =: 4-(diethylamino)-benzaldehyde
GTCS =: general tonic–clonic seizures
het =: heterozygous
NpHR =: halorhodopsin
KI =: knock-in
NREM =: non-rapid eye movement
REM =: rapid eye movement
SWO =: slow-wave oscillation
SWD =: spike–wave discharge
SSWD =: slow SWD
wt =: wild-type
